# Ohio College of Dental Surgery

**Published:** 1883-04

**Authors:** 


### Ohio College of Dental Surgery.

The thirty-seventh annual commencement of this institution was held in College Hall, Cincinnati, Wednesday evening, March 7, 1883, at 8 o'clock.

The annual address was delivered by Prof. L. Buffatt, M.D., D.D.S., of Cleveland, O. And the address on behalf of the class by J. M. Letcher, jr., D.D.S.

The degree of Doctor of Dental Surgery was conferred on the following persons by Dr. C. R. Taft, Vice-President of the Board of Trustees, following which he gave some words of fatherly advice:

| NAMES. | RESIDENCE. |
|---|---|
| James B. Adams, | Kentucky. |
| John M. Brookins, | Indiana. |
| Horatio A. Black, | Ohio. |
| W. H. Craft, | Pennsylvania. |
| Wm. H. Collins, | Ohio. |
| Geo. S. Crider, | Ohio. |
| W. H. Clark, | Illinois. |
| W. C. Duckwall, | Ohio. |
| Edw. P. Green, | Ohio. |
| C. W. Gambrill, | Illinois. |
| Wm. L. Jerman, | Ohio. |
| Jas. H. Letcher, jr., | Kentucky. |
| Augustus E. McConkey, | Ohio. |
| Francis W. Meinhardt, | Wisconsin. |
| Arthur W. Meyer, | Wisconsin. |
| Grant Mollyneaux, | Ohio. |
| Walter D. M. Mason, | Georgia. |
| Geo. W. Ostrander, | Illinois. |
| Chas. F. Oglesbee, | Ohio. |
| Theodore J. Phillips, | Ohio. |
| Jas. J. Rapp, | Ohio. |
| Albert B. Stephens, | Illinois. |
| Sallie Strasburg, | Ohio. |
| Beecher B. Tatman, | Indiana. |
| S. E. Wilhelm, | Iowa. |
| Chas. Whaley, | Ohio. |
| B. Frank Ward, | Ohio. |
| Chas. M. Watson, | Ohio. |

Number of matriculates for the term was fifty-three.

In medical science, we are too apt to rush off after some new theory, just because one of the leading workers has declared his faith in that direction.

